# Supplementary material for: MAVS polymers smaller than 80 nm induce mitochondrial membrane remodeling and interferon signaling
Source: FEBS J. 2019 Feb 16;286(8):1543–60. doi: 10.1111/febs.14772 (PMC6513760; doi:10.1111/febs.14772)
Supplement: Supplementary file 1 — Data S1. IMAGEJ macro script used to analyze mitochondrial distance, area, and length parameters. Data S2. MATLAB code to calculate the effective optical resolution of the STORM images as described by Nieuwenhuizen et al. (ref. 39). Data S3. C++ script used to perform size and cluster analysis of fluorescent foci in the STORM images. [file FEBS-286-1543-s001.zip › febs_14772-sup-0001.pdf]

FEBS PRESS
science publishing by scientists
WILEY
The FEBS Journal
www.febsjournal.org

MAVS polymers smaller than 80 nm induce mitochondrial membrane remodeling and interferon signaling

Ming-Shih Hwang, Jérôme Boulanger, Jonathan D. Howe, Anna Albecka, Mathias Pasche, Leila Muresan and Yorgo Modis

DOI: 10.1111/febs.14772
